# Intracorporeal versus extracorporeal anastomosis in right hemicolectomy: a systematic review and meta-analysis

**DOI:** 10.1007/s00464-016-4982-y

**Published:** 2016-06-10

**Authors:** Stefan van Oostendorp, Arthur Elfrink, Wernard Borstlap, Linda Schoonmade, Colin Sietses, Jeroen Meijerink, Jurriaan Tuynman

**Affiliations:** 1 Department of Surgery, VU University Medical Centre, De Boelelaan 1117, Postbus 7057, 1007 MB Amsterdam, The Netherlands; 2 Department of Surgery, Academic Medical Center, Amsterdam, The Netherlands; 3 Medical Library, VU University Medical Centre, Amsterdam, The Netherlands; 4 Department of Surgery, Gelderse Vallei Hospital Ede, Ede, The Netherlands

**Keywords:** Laparoscopy, Right hemicolectomy, Totally laparoscopic, Laparoscopic assisted, Intracorporeal, Extracorporeal, Anastomosis

## Abstract

**Background:**

Laparoscopic right hemicolectomy for colon cancer is associated with substantial morbidity despite the introduction of enhanced recovery protocols and laparoscopic surgery. Laparoscopic right hemicolectomy with an intracorporeal anastomosis (IA) is less invasive than laparoscopic assisted hemicolectomy, possibly leading to further decrease in post-operative morbidity and faster recovery. The current standard technique includes an extracorporeal anastomosis with mobilization of the colon, mesenteric traction and a extraction wound located in the mid/upper abdomen with relative more post-operative morbidity compared to extraction wounds located in the lower abdomen.

**Methods:**

A systematic review of PubMed and Embase databases was performed on studies comparing the intracorporeal versus the extracorporeal performed anastomosis in laparoscopic right hemicolectomy. Primary outcomes were mortality, short-term morbidity and length of stay. For quality assessment, the MINORS checklist was used. Meta-analysis was performed using a random-effects model, and a subgroup analysis was performed for data regarding short-term morbidity and length of stay in studies published in 2012≥.

**Results:**

A total of 2692 papers were identified, 12 non-randomized comparative studies were included in the analysis with a total number of 1492 patients. No significant change in mortality was found (OR 0.36, 95 % CI 0.09–1.46; *I* ^2^ = 0 %). Short-term morbidity decreased significantly in favour of IA (OR 0.68, 95 % CI 0.49–0.93; *I* ^2^ = 20 %). Length of stay was decreased, but with serious risk of heterogeneity (MD −0.77 days, 95 % CI −1.46 to −0.07; *I* ^2^ = 81 %). Subgroup analysis for papers published in 2012≥ resulted in an even larger decrease in short-term morbidity (OR 0.65, 95 % CI 0.50–0.85; *I* ^2^ = 0 %) and a significant decrease in length of stay with low risk of heterogeneity (MD −0.77 days, 95 % CI −1.17 to −0.37; *I* ^2^ = 4 %).

**Conclusion:**

Intracorporeal anastomosis in laparoscopic right hemicolectomy is associated with reduced short-term morbidity and decreased length of hospital stay suggesting faster recovery as shown in this meta-analysis.

## Background

Colorectal carcinoma is the second most common form of cancer in the western world, with an estimated incidence of 1.36 million cases in 2012 worldwide [1, 2]. Right sided hemicolectomy for right sided colonic cancer is a common performed procedure [3]. Currently, in most countries, the laparoscopic assisted right hemicolectomy with an extracorporeal anastomotic (EA) technique is the standard technique. However, despite introduction of laparoscopic surgery [4] and enhanced recovery protocols [5] in colorectal surgery, morbidity remains substantial. Large randomized trials and national registry data show that the overall in hospital morbidity is still approximately 30 % [3, 5, 6].

Morbidity associated with laparoscopic right hemicolectomy includes prolonged ileus, pain-associated decreased pulmonary function and wound infection leading to subsequent increased length of stay [3, 5, 6]. The current standard procedure for laparoscopic right hemicolectomy includes formation of an extracorporeal anastomosis requiring mobilization of the colon and mesenteric traction in order to extract the ileum and ascending colon theoretically leading to more surgical trauma [7]. Furthermore, the EA technique requires the extraction wound to be located in the mid/upper abdomen with relative more post-operative morbidity compared to a wound in the lower abdomen, since it is known that an incision in the mid/upper abdomen tend to result in increased post-operative pain and compromise pulmonary function compared to lower extraction wounds such as the Pfannenstiel [5, 8].

Recent developments in minimal invasive techniques have facilitated intracorporeal stapled anastomosis (IA). This technique enables a smaller extraction wound in the lower abdominal wall and enables a resection of the right colon with less mobilization and mesenteric traction. Potentially, the risk of mesenteric twisting is less compared to the EA technique [9]. Disadvantages of the intracorporeal anastomosis technique include a longer learning curve and laparoscopic skills including suturing and a risk of intraabdominal faecal spillage [10]. Despite potential benefits of the intracorporeal technique, previous reviews published in 2013 failed to show clear advantages of the newer technique [11, 12]. Since the more recently published studies [13–19] show benefits in short-term morbidity and shorter length of stay for the IA technique, we have conducted an up-to-date systematic review with the most recent studies to investigate the value of the intracorporeal anastomotic technique for laparoscopic right hemicolectomy. We hypothesized that an intracorporeal performed anastomosis leads to a decrease in short morbidity resulting in a shorter length of stay. Secondary endpoints include anastomotic leakage, ileus, incisional surgical site infection and incisional hernia. This systematic review aims to provide a complete overview of studies comparing both techniques.

## Methods

A systematic literature review was performed according to guidelines from the Preferred Reporting Items for Systematic Reviews and Meta-Analyses checklist (PRISMA) [20].

### Search strategy

A comprehensive search was performed in the bibliographic databases PubMed and Embase from inception to 21 December 2015, in collaboration with a medical librarian. Search terms included controlled terms (Mesh in PubMed, Emtree in Embase), as well as free-text terms. The following terms were used (including synonyms and closely related words) as index terms or free-text words: ‘colectomy’, ‘anastomosis’, ‘intracorporeal’, ‘extracorporeal’ and ‘laparoscopy’. The search was performed without date, language or publication status restriction. All titles were screened, and appropriate abstracts were reviewed. See ‘Appendix’ for the search strategy.

### In- and exclusion criteria

Studies eligible for inclusion were: RCT’s, comparative studies on intra- versus extracorporeal anastomosis in laparoscopic right hemicolectomy, and human studies. Exclusion criteria were: non-right hemicolectomy (i.e. transverse or left hemicolectomy, sigmoidectomy, subtotal colectomy), non-comparative (case series, description of technique), single-incision surgery, purely robotic surgery and open hemicolectomy.

### Selection process

After removal of duplicates, two independent reviewers (SvO and AE) selected the studies by screening on title and abstract. If necessary, a third author was consulted in case of disagreement. Two reviewers (SvO and AE) analysed the resulting papers in full text using the online Covidence review manager (Covidence online review manager 2015, www.covidence.org). Further studies were identified by reference checking of the included studies.

### Quality assessment and scoring

To asses methodological quality of the included studies, the ‘Methodological index for non-randomized studies’ (MINORS) instrument was used [21]. We considered follow-up for short-term outcomes as a period 30 days. ‘Follow-up period appropriate to the aim of the study’ was considered reported inadequate if outcomes were not defined as 30-day complications or 30-day readmission rate. The interval of long- or medium-term follow-up (FU) had to be reported explicitly. ‘Loss to follow-up’ was scored with 2 points if mentioned explicitly or if it could be derived from the outcomes (i.e. percentage 30-day readmission). If end of the FU-period was not yet achieved in all patients, ‘Loss to follow-up’ was rewarded 1 point. Prospective collection of data was adequately reported if the authors explicitly mentioned the use of a prospectively maintained database.

### Outcomes of interest

Our primary outcomes of interest were short-term morbidity, mortality and length of stay. Secondarily, we looked at the intraoperative outcomes and the rates of anastomotic leak rate, ileus, incisional surgical site infection (SSI) and incisional hernia. Because the definitions of short-term morbidity varied among the included studies, we derived short-term morbidity of each study separately. If the Clavien–Dindo classification for post-operative complications was used, class V (death) was separated from the total of complications to assess mortality. SSI was considered to be a superficial or deep incisional wound infection, but not as an intraabdominal abscess or organ space infection. Incisional hernia was specified to the extraction site and did not include trocar site herniation. It was postulated that the learning curve of the surgeons could have an impact on the outcomes of the IA. Therefore, a subgroup analysis was performed for studies published in 2012 and later on short-term morbidity and length of stay to see whether the more recent studies showed a larger effect.

### Quantitative analysis

Data analysis was performed with the use of Revman 5.0 (Review Manager 5.0, Copenhagen, Denmark: The Nordic Cochrane Centre, The Cochrane Collaboration, 2008). Dichotomous outcomes were statistically analysed and summarized by using the odds ratio (OR) with a confidence interval (CI) of 95 %. Mantel–Haenszel method was used to combine the OR of the outcomes using a random-effects model. Continuous outcomes were analysed by computing a mean difference (MD). OR < 1 favours the IA group and was considered statistically significant if *p* < 0.05 if the 95 % CI did not include 1. Heterogeneity was assessed by performing an *I* ^2^ statistic and a Chi-squared test, considering *I* ^2^ > 50 % and Chi-squared *p* value <0.1 as statistically significant heterogeneity [22]. A subgroup analysis was done for data regarding short-term morbidity and length of stay in studies published in 2012≥.

## Results

### Literature search

**Fig. 1:**
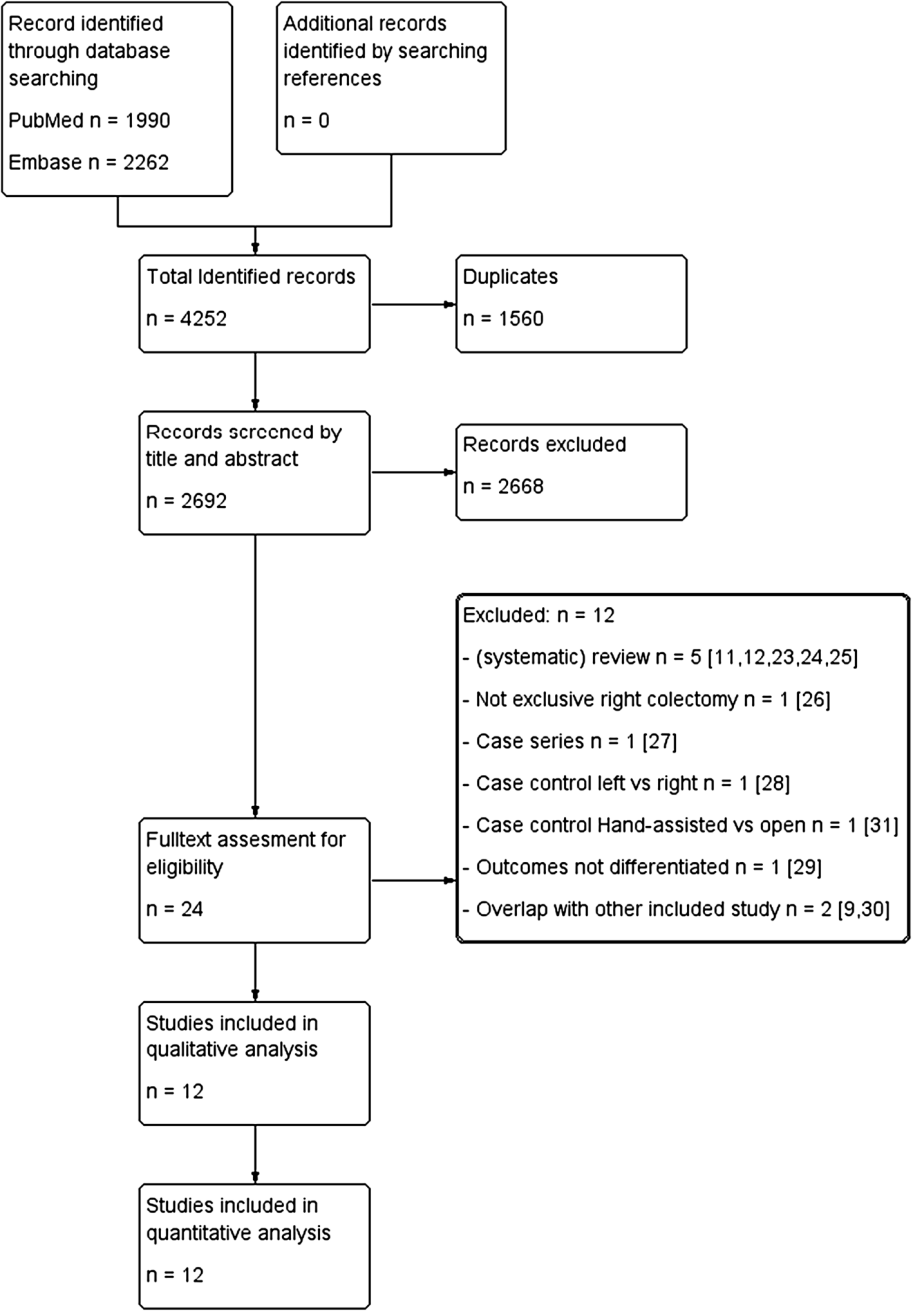
Flowchart

The search resulted in a total of 2692 papers after removal of duplicates. After screening on title and abstract, 24 papers were assessed by full text. A total of 12 papers were excluded for various reasons [9, 11, 12, 23–31], see Fig. 1. Finally, 12 studies were incorporated in the qualitative analysis [13–19, 32–36]. For studies with overlap, we included the most recent publications which consisted of more patients [13, 36] and excluded the earlier studies [9, 23]. No additional studies were identified by cross-checking the references of the included papers.

Magistro et al. reported the only prospective study that alternatively assigned patients to the two procedures [14]. Eleven studies were of retrospective design [13, 15–19, 32–36]. Milone et al. [16] matched the control group using a propensity score. Trastulli et al. [18] reported a retrospective multicenter case series on right colectomy comparing robotic intracorporeal anastomosis to laparoscopic intracorporeal anastomosis and laparoscopic extracorporeal anastomosis. The included studies resulted in a total number of 1492 participants who underwent a laparoscopic right hemicolectomy further specified to 763 and 729 patients for intra- or extracorporeal anastomosis, respectively. Study design and patient characteristics are described in Table 1. In nine studies, the intracorporeal performed anastomosis was created using a mechanical stapler with [13–15, 17–19, 33, 35, 36] or without [16] additional sutures in the IA technique. A mechanical stapler was most commonly used for the extracorporeal anastomosis as well (with [13, 14, 18, 36] or without [16, 19] additional sutures). One study made a hand-sewn anastomosis [15] or according to the preference of the individual surgeon (mechanical or hand-sewn) [17, 33]. Two studies did not specify the creation of the anastomosis [32, 34].

**Table 1:** Study characteristics

Study (author, YoP)	Design	Malignant	Benign	Patients (*n*)		Age		BMI (kg/m^2^)		ASA classification	
				IA	EA	IA	EA	IA	EA	IA	EA
Anania, 2012	Retrospective CCS	+	−	39	33	74.5 (53–89)^b^	74 (45–96)^b^	26.3 (20–37)^b^	28.1 (19.9–37)^b^	NR	NR
Chaves, 2011	Retrospective CCS	+	+	35	25	62.6 (13.4)^a^	58.9 (12.9)^a^	25.9 (3.1)^a^	26.7 (3.9)^a^	17/18^c^	15/10^c^
Fabozzi, 2010	Retrospective CCS	+	−	50	50	62.1 (8.3)^a^	59.4 (9.5)^a^	21.4 (2.3)^a^	22.1 (1.6)^a^	2 (1–2)^b^	2 (1–2)^b^
Lee, 2013	Retrospective CCS	+	+	51	35	70 (43–90)^b^	66 (48–93)^b^	25.7 (18–46.5)^b^	25.4 (18.3–45.3)^b^	3 (2–4)^b^	3 (1–3)^b^
Magistro, 2013	Prospective CCS	+	+	40	40	70.9 (13.4)^a^	71.2 (10.5)^a^	24.8 (2.8)^a^	23.9 (4.4)^a^	2 (1–3)^b^	2 (1–3)^b^
Marchesi, 2013	Retrospective CCS	+	+	28	27	66.2^a^	67.7^a^	26.1^a^	26.2^a^	19/9^c^	17/10^c^
Milone, 2015	Retrospective CCS	+	+	286	226	67.7 (12.6)^a^	65.6 (11.4)^a^	25.2 (3.8)^a^	25.4 (3.8)^a^	2 (1–4)^b^	2 (1–4)^b^
Roscio, 2012	Retrospective CCS	+	−	42	30	63.5 (10.3)^a^	63.7 (10.3)^a^	26.0 (4.0)^a^	26.3 (3.8)^a^	2 (1–3)^b^	2 (1–3)^b^
Scatizzi, 2010	Retrospective CCS	+	−	40	40	70 (47–87)^b^	68.5 (41–85)^b^	28^a^	27^b^	2 (1–3)^b^	2 (1–3)^b^
Shapiro, 2015	Retrospective CCS	+	−	91	100	72 (45–90)^b^	72 (49–90)^b^	27.8 (4.6)^a^	26.9 (4.3)^a^	3 (1–4)^b^	3 (1–4)^b^
Trastulli, 2015	Retrospective CCS	+	+	40	94	71.5^a^	70.8^a^	26.6^a^	25.4^a^	2 (1–3)^b^	2 (1–3)^b^
Vergis, 2015	Retrospective CCS	+	+	21	29	65^a^	69^a^	27^a^	28^a^	2.65^a^	3.04^a^

*YoP* year of publication, *CCS* case-controlled series, *ASA* American Society of Anaesthesiologists, *N* number

^a^Mean (SD), ^b^ median (range), ^c^ ASA-score 1 + 2/3 + 4, number of patients

### Quality assessment: MINORS instrument

**Fig. 2:**
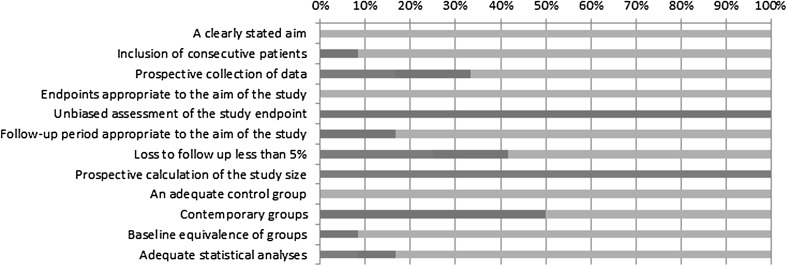
MINORS quality assessment

The quality assessment is shown in Table 2 and Fig. 2. The mean score was 18.8 (range 16–21) out of a total of 24 points. In some studies reporting on mid- or long-term outcomes, the foreseen follow-up period was not achieved in all patients and was regarded as reported but defined as ‘not adequately’ [13, 17]. Several studies, aiming to compare short-term outcomes, failed to (adequately) report 30-day outcomes including readmission and/or reported no visits to the outpatient clinic after discharge [14, 16, 32]. Interestingly, Scatizzi et al. [36] defined short-term outcomes as 90 days and reported an outpatient clinic visit 8 days after discharge, but subsequently failed to report on the 3 month FU besides readmission. Half of the studies changed their way of operation halfway during the score inclusion period from EA to IA, using their last EA as ‘historic’ control group [15, 18, 19, 32, 33, 35]. All studies scored low on unbiased assessment of outcomes due to lack of blinding and randomization. None calculated a sample size since 11 studies were retrospective and 1 study was only pseudo-randomized [14].

**Table 2:** MINORS quality assessment

	A clearly stated aim	Inclusion of consecutive patients	Prospective collection of data	Endpoints appropriate to the aim of the study	Unbiased assessment of the study endpoint	Follow-up period appropriate to the aim of the study	Loss to follow-up less than 5 %	Prospective calculation of the study size	An adequate control group	Contemporary groups	Baseline equivalence of groups	Adequate statistical analyses	Total
Anania	*2*	*2*	**1**	*2*	**1**	*2*	***0***	***0***	*2*	**1**	*2*	*2*	17
Chaves	*2*	*2*	*2*	*2*	**1**	*2*	*2*	***0***	*2*	**1**	*2*	*2*	20
Fabozzi	*2*	**1**	***0***	*2*	**1**	*2*	*2*	***0***	*2*	*2*	*2*	**1**	17
Lee	*2*	*2*	***0***	*2*	**1**	**1**	*2*	***0***	*2*	*2*	*2*	*2*	18
Magistro	*2*	*2*	*2*	*2*	**1**	*2*	***0***	***0***	*2*	*2*	*2*	*2*	19
Marchesi	*2*	*2*	*2*	*2*	**1**	*2*	*2*	***0***	*2*	**1**	*2*	*2*	20
Milone	*2*	*2*	*2*	*2*	**1**	*2*	**1**	***0***	*2*	2	*2*	*2*	20
Scatizzi	*2*	*2*	*2*	*2*	**1**	*2*	*2*	***0***	*2*	*2*	*2*	*2*	21
Shapiro	*2*	*2*	*2*	*2*	**1**	**1**	*2*	***0***	*2*	*2*	*2*	*2*	20
Roscio	*2*	*2*	*2*	*2*	**1**	*2*	**1**	***0***	*2*	**1**	*2*	*2*	19
Trastulli	*2*	*2*	*2*	*2*	**1**	*2*	**1**	***0***	*2*	**1**	**1**	***0***	16
Vergis	*2*	*2*	**1**	*2*	**1**	*2*	*2*	***0***	*2*	**1**	*2*	*2*	19
Not reported	0	0	2	0	0	0	2	12	0	0	0	1	17
Reported, inadequate	0	1	2	0	12	2	3	0	0	6	1	1	28
Reported, adequate	12	11	8	12	0	12	7	0	12	3	11	10	98

Percentage adequately reported (italics). Percentage reported but inadequate: 1 point (bold). Percentage not reported: 0 points (bold italics).

### Primary outcomes

#### Mortality

**Fig. 3:**
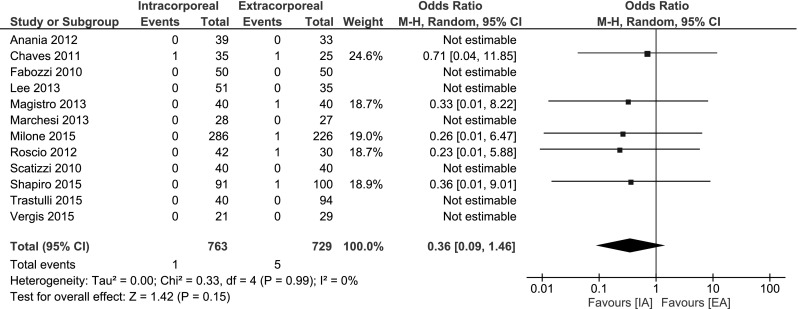
Mortality

No significant difference in mortality was observed for both procedures: OR 0.36, 95 % CI 0.09–1.46; *I* ^2^ = 0 % (Fig. 3).

#### Short-term morbidity

**Fig. 4:**
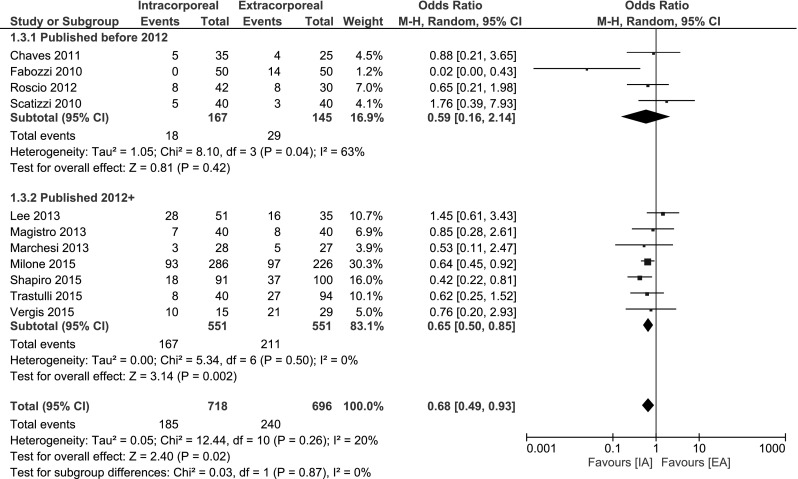
Short-term morbidity

A significant decrease in short-term morbidity was observed when performing an IA: OR 0.68, 95 % CI 0.49–0.93; *I* ^2^ = 20 %. Subgroup analysis on studies published ≥2012 showed a larger decrease and less risk at heterogeneity: OR 0.65, 95 % CI 0.50–0.85; *I* ^2^ = 0 %. Four studies reported morbidity according to Clavien–Dindo [15–17, 35]. Two other studies reported 30-day complication rate [18, 33]. One study described the amount of complications in text [32]. The remaining studies provided a table of complications differentiated to mortality, minor and major morbidity [13, 14, 34, 36] (Fig. 4).

#### Length of stay

**Fig. 5:**
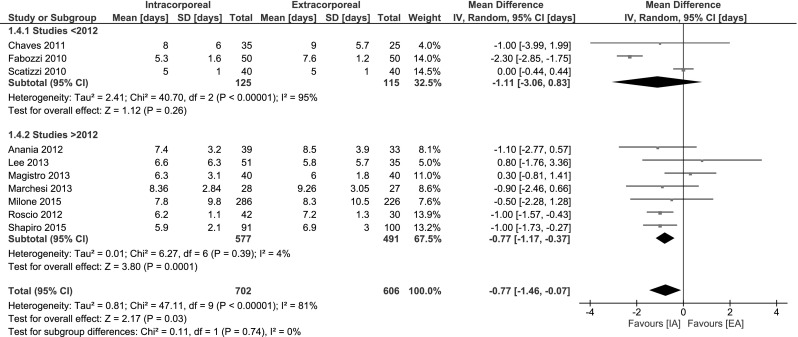
Length of stay

In the meta-analysis, Length of stay (LoS) was significantly decreased if favour of IA: MD −0.77 days, 95 % CI −1.46 to −0.07. However, heterogeneity among studies was substantial. Subgroup analysis on studies published ≥2012 was more homogenous and showed a statistically significant decrease in LoS (0.77 days, 95 % CI −1.17 to −0.37) (Fig. 5). Two studies were not included in the meta-analysis. Trastulli et al. [18] provided a median (range) of 5.5 days (3–14) for IA versus 7 (4–21) in the EA group. The mean LoS in the study by Vergis et al. [19] was 5.33 and 5.86 for IA and EA, respectively. Unfortunately, no SD was provided.

### Secondary outcomes

#### Intraoperative outcomes

##### Duration of surgery

Operating time varied widely, with conflicting significant outcomes in either IA or EA. Magistro et al. and Shapiro et al. reported a significant longer duration of surgery (DoS) for IA [14, 17]. In contrast, Fabozzi et al. [34] and Roscio et al. [35] stated the IA technique was faster. However, most studies showed no significant difference. Interestingly, Marchesi et al. reported the time to perform the anastomosis separately and showed an impressive reduction at the end of his IA series indicating a learning curve. The mean DoS of his last 10 IA was 161 min versus his mean EA time of 186.8 min [15]. See ‘Appendix’.

#### Post-operative outcomes

##### Anastomotic leak rate

**Fig. 6:**
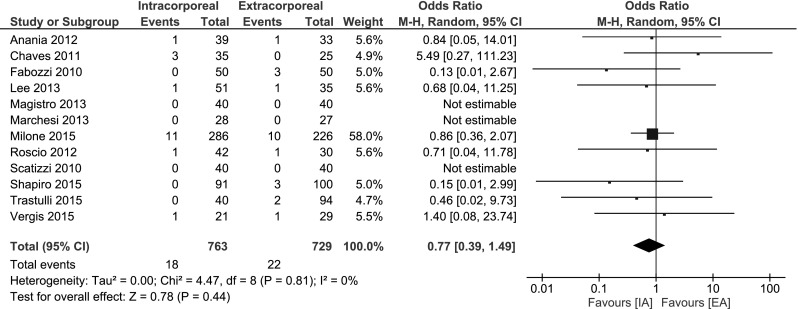
Anastomotic leak

No statistically significant difference between the IA or EA technique was found for anastomotic leakage: OR 0.77, 95 % CI 0.39–1.49; *I* ^2^ = 0 % (Fig. 6).

##### Ileus

**Fig. 7:**
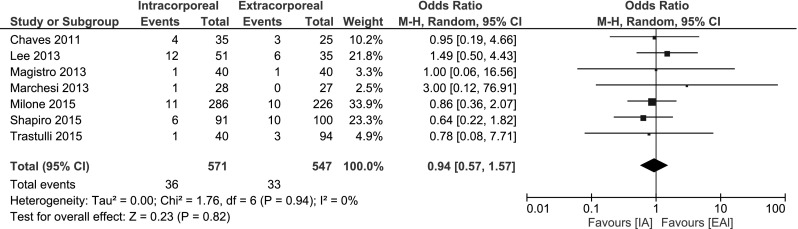
Ileus

The incidence of an ileus was reported in 6 studies [13–18, 33], no significant change was found: OR 0.94, 95 % CI 0.57–1.57; *I* ^2^ = 0 % (Fig. 7).

##### Surgical site infection

**Fig. 8:**
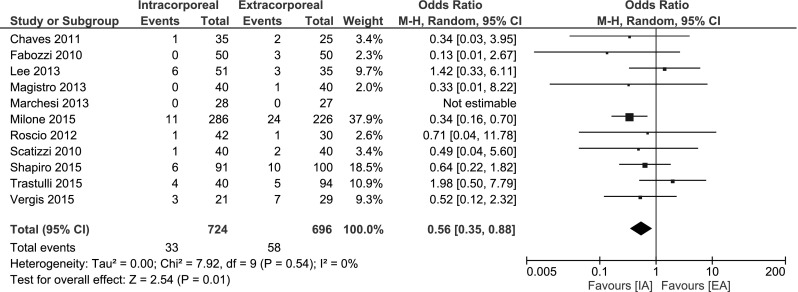
Surgical site infection

All but one study [32] mentioned the occurrence of a surgical site infection (please note: superficial and deep incisional surgical site infection, not abscess or organ spaced SSI). A significant decrease in SSI was found (OR 0.56, 95 % CI 0.35–0.88; *I* ^2^ = 0 %.) in favour of IA (Fig. 8).

##### Incisional hernia

Five studies reported incisional hernia, see ‘Appendix’. No meta-analysis was performed since follow-up and extraction technique varied. For instance, all the hernia’s in the IA group by Shapiro (*n* = 2) and Chaves (*n* = 2) was observed in patients having had an extraction other than the routinely performed Pfannenstiel incision.

##### Return of bowel function

There was a variety in reporting on return of bowel function among included studies. Four studies [32, 33, 35, 36] showed significant earlier bowel movement in the IA group, and two different papers [14, 16] reported significant earlier first flatus pointing towards an sooner return of bowel function for IA. See ‘Appendix’.

## Discussion

This systematic review comparing intracorporeal versus extracorporeal anastomosis in laparoscopic right hemicolectomy shows that the intracorporeal technique is associated with significant decreased short-term morbidity and length of stay. No differences were observed for mortality, Ileus and anastomotic leakage. In a subgroup analysis of the more recent studies (2012≥), the observed differences were larger with less heterogeneity in favour of IA.

The observed decreased morbidity of the intracorporeal anastomosis technique seems largely related to the extraction site. By performing an IA, the incision for specimen extraction can be smaller and the incision can be performed in the lower part of the abdomen, which has shown to be associated with less pain, less pulmonary morbidity, a lower infection rate and on the long-term lower herniation rate [37, 38]. The suprapubic (Pfannenstiel) site for specimen extraction is the preferred extraction site since it has been reported to be associated with low site infections and with a low hernia rate of only 0–2 % [39]. Shapiro et al. [17] found such hernia rates in their series (IA 2.2 %, EA 17 %). The 2 hernia’s in the IA were not Pfannenstiel incisions but periumbilical and midline. Chaves et al. [33] report 2 versus 1 hernia in IA and EA, respectively. However, again these two cases in the IA-arm were not extracted by a Pfannenstiel incision, but a midline incision was chosen since both patients had a previous laparotomy. Furthermore, IA requires a smaller incision potentially leading to less post-operative pain [40] with a possible reduction in hospital costs [41], shorter hospital stay [4] and pulmonary dysfunction [8]. The observed decreased morbidity in the IA group might also be related to less mobilization of the transverse colon and less traction on the mesentery and pancreatico-duodenal block, theoretically resulting in surgical trauma and earlier restoration of bowel function [19, 35]. Especially, in obese patients, the mesentery is subject to substantial traction to externalize the bowel in EA [12, 25, 33, 42, 43].

Total mortality did not statistically differ. Short-term morbidity was significantly decreased in favour of IA. This advantage was even larger for the more recent studies as shown after subgroup analysis. The length of stay seems shorter; however, this was not significant. In addition, serious risk at heterogeneity was observed in the meta-analysis, so no conclusions can be made. However, subgroup analysis of the recent studies did reveal an significant decrease in LoS in favour of IA as is expected since the morbidity is less. See Fig. 5.

Incisional SSI was significantly decreased when an IA was performed. Some authors discussed that externalizing the bowel in EA requires more traction and tension of the wound resulting in more tissue trauma [26]. No significant differences in anastomotic leak and ileus rate were found. In contrast, using IA technique, the necessity for intraperitoneal tomies into the contaminated transversum and ileum could lead to a theoretical increase of intraabdominal infections. Chang et al. [44] described the use of atraumatic intracorporeal bulldogs to minimize faecal spillage when performing an IA. Since the included studies heterogeneously reported on intraabdominal abscesses and/or interventions, we cannot conclude that the IA has a significant influence on deep abdominal abscesses compared to standard EA.

Potential new techniques for extraction include transvaginal colectomy, a form of natural orifice specimen extraction (NOSE). This might even further decrease surgical trauma, although large cohort data and randomized evidence is lacking [45]. Nevertheless, small cohort series show promising results for partial colectomy with minor short-term morbidity and a shorter length of stay [45, 46]. For male, transgastric or transrectal extraction creates potential more surgical trauma, and a small Pfannenstiel is still considered as the best option. Currently, the available data are insufficient to make any statements regarding safety and efficacy of natural orifice transluminal endoscopic surgery (NOTES) for laparoscopic right hemicolectomy.

This systematic review and meta-analysis has several limitations. The included studies are merely observational, and the majority (*n* = 11 out of 12) was of retrospective design. Complications according to Clavien–Dindo classification were reported only in 25 % of the included studies. Studies focused merely on short-term outcomes and reported corresponding follow-up. As we foresee, a considerable reduction in the incidence of incisional hernia following IA technique, and longer follow-up (i.e. 2 years) would provide more insight [37].

## Conclusion

This meta-analysis of non-randomized, comparative studies shows that intracorporeal anastomosis in laparoscopic right hemicolectomy is associated with reduced short-term morbidity and decreased length of hospital stay suggesting faster recovery. A randomized controlled trial is warranted to confirm these findings.

## Appendix

See Tables 3, 4, 5, 6, 7 and 8.

**Table 3:** PubMed search 21 December 2015

	PubMed search 21 December 2015	*N*
#1	‘Colectomy’[Mesh:NoExp] OR colectom*[tiab] OR hemicolectom*[tiab] OR colon resection*[tiab] OR colorectal resection*[tiab] OR large bowel resection*[tiab]	21,875
#2	‘Anastomosis, Surgical’[Mesh:NoExp] OR anastom*[tiab]	80,578
#3	intracorpo*[tiab] OR intra-corpo*[tiab] OR intra-abdom*[tiab] OR intraabdom*[tiab] OR ICA[tiab] OR extracorpo*[tiab] OR extra-corpo*[tiab] OR extra-abdom*[tiab] OR extraabdom*[tiab] OR ECA[tiab]	71,900
#4	((‘Laparoscopy’[Mesh:NoExp] OR laparoscop*[tiab]) AND (total*[tiab] OR assisted[tiab]))	24,110
#5	#2 AND #3	2756
#6	#4 or #5	26,407
#7	#1 AND #6	1990

**Table 4:** Embase search 21 December 2015

	Embase search 21 December 2015	*N*
#1	‘colon resection’/de OR **’hemicolectomy’**/exp OR **colectom***:ab,ti OR **hemicolectom***:ab,ti OR (**colon** NEAR/3 **resection***):ab,ti OR (**colorectal** NEAR/3 **resection***):ab,ti OR (**‘large bowel’** NEAR/3 **resection***):ab,ti	42,437
#2	‘anastomosis’/exp OR **anastom***:ab,ti	191,035
#3	intracorpo*:ab,ti OR (**intra** NEAR/3 **corpo***):ab,ti OR (**intra** NEAR/3 **abdom***):ab,ti OR **intraabdom***:ab,ti OR **ica**:ab,ti OR **extracorpo***:ab,ti OR (**extra** NEAR/3 **corpo***):ab,ti OR (**extra** NEAR/3 **abdom***):ab,ti OR **extraabdom***:ab,ti OR **eca**:ab,ti	93,326
#4	laparoscopy’/exp OR **laparoscop***:ab,ti AND (**total***:ab,ti OR **assisted**:ab,ti)	39,455
#5	#2 AND **#3**	5822
#6	#4 OR **#5**	44,350
#7	#1 AND **#6**	3676
#8	#7 AND (**‘article’**/it OR **’article in press’**/it OR **’conference paper’**/it OR **’review’**/it)	2262

**Table 5:** Duration of surgery

Study (author, YoP)	Duration of surgery (min)		
	IA	EA	*p*
Anania, 2012	186.8 (105–280)^c^	184.1 (115–285)^c^	0.6549
Chaves, 2011	227 (44.5)^a^	203 (36.4)^a^	NR
Fabozzi, 2010	78 (25)^a^	92 (22)^a^	**<0.05**
Lee, 2013	205 (132)^a^	196 (56)^a^	NR
Magistro, 2013	230 (45)^a^	203 (48)^a^	**0.011**
Marchesi, 2013	205.79 (45.77)^a^	196.78 (22.95)^a^	0.3952
Milone, 2015	166.9 (10.7)^a^	157.5 (67.2)^a^	0.06
Roscio, 2012	176.5 (40.0)^a^	186.3 (40.1)^a^	0.039
Scatizzi, 2010	150 (115–180)^b^	150 (105–245)^b^	0.167
Shapiro, 2015	155 (37)^a^	142 (35)^a^	**0.006**
Trastulli, 2015	204.3 (51.9)^a^	208 (61)^a^	NR
Vergis, 2015	170 (121–237)^b^	181 (98–205)^b^	0.78

Bold values are statistically significant (*p* < 0.05)

*YoP* year of publication, *Min* minutes, *N* number, *NR* not reported

^a^Mean (SD), ^b^ median (range), ^c^ mean (range)

**Table 6:** Incisional hernia

Study (author, YoP)	Hernia *n* (%)		
	IA	EA	*p*
Anania, 2012	NR	NR	–
Chaves, 2011	2 (5.7)	1 (4)	–
Fabozzi, 2010	NR	NR	–
Lee, 2013	1 (1.9)	3 (8.6)	–
Magistro, 2013	NR	NR	–
Marchesi, 2013	NR	NR	–
Milone, 2015	NR	NR	–
Roscio, 2012	0	1 (3.3)	–
Scatizzi, 2010	NR	NR	–
Shapiro, 2015	2 (2.2)	17 (17.0)	**0.001**
Trastulli, 2015	NR	NR	–
Vergis, 2015	0	6 (20.7)	**0.026**

Bold values are statistically significant (*p* < 0.05)

*YoP* year of publication, *N* number, *NR* not reported

**Table 7:** Return of bowel function

Study (author, YoP)	Bowel movement (days)			First flatus (days)		
	IA	EA	*p*	IA	EA	*p*
Anania, 2012	3.8 (1.4)^a^	4.9 (1.5)^a^	**<0.0001**	NR	NR	–
Chaves, 2011	3 (2–8)^b^	4 (2–8)^b^	**0.004**	NR	NR	–
Fabozzi, 2010	3.1 (1.2)^a^	4.4 (1.6)^a^	NS	NR	NR	–
Lee, 2013	NR	NR	–	NR	NR	–
Magistro, 2013	3.5 (1.1)^a^	3.8 (1.1)^a^	0.234	2.2 (0.6)^a^	2.6 (0.8)^a^	**0.043**
Marchesi, 2013	NR	NR	–	NR	NR	–
Milone, 2015	NR	NR	–	1.7 (1)^a^	2.3 (0.8)^a^	**<0.001**
Roscio, 2012	2.9 (0.9)^a^	3.4 (0.9)^a^	**0.023**	NR	NR	–
Scatizzi, 2010	0 (0–1)^b^	1 (0–1)^b^	**0.043**	NR	NR	–
Shapiro, 2015	NR	NR	–	NR	NR	–
Trastulli, 2015	NR	NR	–	4 (1–7)^b^	3 (1–6)^b^	–
Vergis, 2015	NR	NR	–	NR	NR	–

Bold values are statistically significant (*p* < 0.05)

*YoP* year of publication, *Min* minutes, *N* number, *NR* not reported

^a^Mean (SD), ^b^ median (range)

**Table 8:** Incision length and tolerance to solid diet

Study (author, YoP)	Incision length			Tolerance to solid diet (days)		
	IA	EA	*p*	IA	EA	*p*
Anania, 2012	NR	NR	–	4.6 (2.1)^a^	5.7 (1.7)^a^	**<0.0001**
Chaves, 2011	NR	NR	–	1 (1–9)^b^	2 (1–10)^b^	**0.002**
Fabozzi, 2010	6.0 (1)^a^	12.0 (2)^a^	**<0.05**	NR	NR	–
Lee, 2013	NR	NR	–	NR	NR	–
Magistro, 2013	5.5 (1.1)^a^	7.2 (1.3)^a^	**0.01**	NR	NR	–
Marchesi, 2013	4.8 (0.9)^a^	7.2 (1.1)^a^	**0.02**	NR	NR	–
Milone, 2015	NR	NR	–	NR	NR	–
Roscio, 2012	NR	NR	–	NR	NR	–
Scatizzi, 2010	4.0 (3.0–7.0)^b^	5.0 (3.0–7.0)^b^	**0.019**	1 (1–8)^b^	2 (1–12)^b^	**0.025**
Shapiro, 2015	NR	NR	–	NR	NR	–
Trastulli, 2015	NR	NR	–	NR	NR	–
Vergis, 2015	NR	NR	–	2.34^a^	3.21^a^	**0.023**

Bold values are statistically significant (*p* < 0.05)

*YoP* year of publication, *Min* minutes, *N* number, *NR* not reported

^a^Mean (SD), ^b^ median (range)
